# An analysis of the registration of essential medicines in Namibia: a proxy measure of the availability of essential medicines

**DOI:** 10.1080/20523211.2026.2643322

**Published:** 2026-03-23

**Authors:** Anastasia N. Aluvilu, Justice E. K. Sheehama, Saren N. Shifotoka, Juliet Nabyonga-Orem, Petra Brhlikova

**Affiliations:** a World Health Organization, UN House, Windhoek, Namibia; b Namibia Medicines Regulatory Council (NMRC), Windhoek, Namibia; c Centre for Health Professions Education, Faculty of Health Sciences, North-West University, Potchefstroom, South Africa; d Population Health Sciences Institute, Newcastle University, Newcastle upon Tyne, UK

**Keywords:** Essential medicines, registration, availability, AWaRe classification

## Abstract

**Background:**

Many African countries, including Namibia, face challenges with the availability of essential medicines, impacting health outcomes. This study aimed to evaluate the availability of essential medicines in Namibia by examining the registration of medicines on the Namibia Essential Medicines List (NEMList) as a proxy.

**Methods:**

An in-depth analysis of the registration of essential medicines was carried out by comparing medicines in the NEMList against registered medicines on the Namibian Human Medicines Register as of February 2024. Microsoft Excel was used to capture details about the generic names of medicines, strengths, and dosage forms in which the medicines were available. A sub-analysis of antibiotics, which classified them using the Access, Watch and Reserve (AWaRe) classification, was also conducted.

**Results:**

A high proportion of essential medicines listed in the NEMList, 80.2% (386/481) are registered in the country. However, essential medicines only accounted for 23.7% (1,288/5,444) of all medicines on the Namibian Human Medicines Register. Among the essential medicines registered in Namibia, only 45.5% (176/386) had at least three registered products. Of the 55 antibiotics on the NEMList, 94.5% (52/55) had registered products, with 96.2% (25/26), 92.0% (23/25) and 100% (4/4) of Access, Watch and Reserve group antibiotics having registered products, respectively. Despite the high proportions of registered essential antibiotics, only 65.4% of Access group antibiotics, 68.0% of Watch group antibiotics and 25.0% of Reserve antibiotics had at least three registered products.

**Conclusion:**

Registration of essential medicines is crucial to ensure access and availability. Further studies are needed to understand the bottlenecks encountered by the Namibia Medicines Regulatory Council (NMRC) in registering essential medicines. Concerted programmes and policies to strengthen regional harmonisation of medicines registration should be developed, and essential medicines should be prioritised.

## Background

Access to safe, effective, and quality-assured medicines is a cornerstone of public health. The availability of essential medicines is a significant indicator of a functional healthcare system and is critical to improve the health outcomes of patients. According to the World Health Organization (WHO), essential medicines are those that ‘satisfy the priority health care needs of the population’ (World Health Organization, 2023a), and should be available at all times, in appropriate dosage forms of assured quality and at an affordable price (World Health Organization, 2023a; Ooms et al., 2023). Essential medicines are uniquely selected based on disease prevalence and public health relevance, evidence of efficacy and safety, and comparative cost-effectiveness (World Health Organization, 2023a).

Over the years, WHO has made significant strides to ensure access to essential medicines in healthcare facilities at all times for treating priority healthcare needs. Notably, this includes the publication of the essential medicines list (EML), which was first released in 1977 to prioritise a limited list of life-saving medicines for treating the healthcare needs of the majority of the population (Yenet et al., 2023). The WHO model list of essential medicines is released biannually and serves as a guide for nearly 137 countries worldwide to develop their own national essential medicines lists based on local health needs and inform treatment guidelines to improve access to quality medicines, promote rational prescribing practices and reduce healthcare costs (Broccoli et al., 2018; Green et al., 2023; World Health Organization, 2023a). Moreover, WHO has designated access to safe and effective medicines as a basic human right (Adebisi et al., 2022).

In 2015, the United Nations General Assembly adopted the Sustainable Development Goals (SDGs), which included ensuring ‘access to safe, effective, quality and affordable essential medicines and vaccines for all’ as part of Target 3.8 (Lane et al., 2024). However, despite these advancements and declarations, the unaffordability and non-availability of essential medicines continue to be one of the most pressing challenges confronting African countries, and they are a major barrier to the provision of quality healthcare services, thus negatively affecting health equity and outcomes for patients in the region (Kibirige et al., 2022; Lane et al., 2024; Yenet et al., 2023).

Market authorisation or registration of essential medicines by National Regulatory Authorities (NRAs) is the first step to ensuring both access and availability at country level (Lyus et al., 2020), yet the extent to which essential medicines are registered is not known for most countries (Brhlikova et al., 2020). Adequate registration of essential medicines by NRAs is imperative to meet public health needs. While there is no published research into the ideal number of registered products for each International Non-proprietary Name (INN) to ensure availability (Green et al., 2023), WHO recommends at least three products registered per the INN by different manufacturers to ensure a stable supply (World Health Organization, 2016).

In Namibia, medicines availability is governed by a national regulatory framework established under the Medicines and Related Substances Control Act (Act No. 13 of 2003), which mandates the Namibia Medicines Regulatory Council (NMRC) as the authority responsible for medicine registration and oversight (Namibia Medicines Regulatory Council, 2024a). As stipulated in Sections 18 and 19 of the Act, all medicines intended for sale, distribution, or use must be registered with the NMRC, except otherwise authorised under Section 27, which allows for special authorisation of unregistered products under exceptional circumstances (Republic of Namibia, 2003). This system reflects global regulatory norms, including the World Health Assembly Resolution 67.20, which calls for medical products’ approval by the national or regional regulatory authorities, in line with current international standards (World Health Organization, 2014). The NMRC is further empowered by the National Medicines Policy (NMP) to ensure the availability of essential medicines by regulating pharmaceutical products through marketing authorisation, inspecting and licencing pharmaceutical manufacturers, wholesalers and distributors, controlling the quality of medicines through quality surveillance testing and providing post-registration surveillance or pharmacovigilance services (Ministry of Health and Social Services, 2022).

The Ministry of Health and Social Services’ Central Medical Stores (CMS) is responsible for pharmaceutical procurement and distribution, to ensure consistent and reliable availability of essential medicines to the public healthcare sector, which caters to 85% of the country’s population (German Federal Ministry for Economic Cooperation and Development (BMZ), 2023). The procurement process is guided by the NEMList, where only medicines included in the NEMList are available to the public health facilities. Procurement of essential medicines by the Central Medical Stores is further guided by the registration status of medicines on the Namibian Human Medicines Register (Ministry of Health and Social Services, 2021). As such, the registration of essential medicines functions as a key determinant of access to medicines for patients at different levels of public health care. Moreover, the registration of at least three products per active ingredient by different manufacturers is pivotal to ensure that essential medicines are available at all times (World Health Organization, 2016).

Despite the systems available to ensure availability to essential medicines, Namibia regularly experiences medicine shortages (Rennie et al., 2020), posing a significant threat to public health. Little is currently known about the availability of essential medicines in Namibia.

The objective of this study was to conduct an audit of registered products on the Namibian Human Medicines Register and compare it with the Namibia Essential Medicines List (NEMList) to establish a proxy measure of the availability of essential medicines in the country. Green et al. (2023) argue that the non-registration of medicines is a barrier to availability (Green et al., 2023). To our knowledge, this is the first study to examine the registration of essential medicines in Namibia.

## Methods

### Study design

This is a retrospective analysis of the registration of essential medicines and medicinal products on the NEMList as of February 2024. The methodology was developed based on a thorough literature review of similar studies conducted in different settings, with necessary modifications to align with the context of Namibia (Babatunde et al., 2024; Brhlikova et al., 2020; Lyus et al., 2020).

### Data sources and extraction

#### The essential medicines list

The NEMList seventh edition launched in 2021 contains 503 products arranged broadly into fourteen main sections according to the international Anatomical Therapeutic Chemical (ATC) classification system (Ministry of Health and Social Services, 2021) and 95 Therapeutic Classes. The medicines in the NEMList are described in six (6) columns including the generic name of the medicine (Active Pharmaceutical Ingredient), Strength, Dosage Form, Class as VEN (Vital, Essential, and Necessary), Central Medical Stores (CMS) catalogue number, and level of healthcare the medicines can be prescribed (Ministry of Health and Social Services, 2021).

Twenty-two products listed in the NEMList that are not regulated by NMRC were excluded from the analysis. These were non-pharmacological products (Diagnostic Agents and Other Non-Therapeutic Products) such as blood glucose and urine test strips, formula powder milk, ECG gel, soda lime with indicator powder, Ready-to-use Therapeutic Foods (RUTF), and Semi Elemental Adult Feeds. After applying these exclusions, a total of 481 products remained for analysis. The following information on medicines from the NEMList was extracted: generic name of the medicine (i.e. as INN or combination of INNs), strength, and dosage form. This was captured into a Microsoft Excel database. Medicines that are available in different dosage forms (e.g. tablets and syrup) were listed in different rows.

#### The Namibian Human Medicines Register

The Namibian Human Medicines Register of February 2024 (Namibia Medicines Regulatory Council, 2024b) listed 5,444 products registered for the Namibian market. The medicines in the Namibian Human Medicines Register are listed based on the Applicant, Proprietary Name of the Medicine, Approved Name of Active Pharmaceutical Ingredient(s) (as single or fixed-dose combination), Dosage Form, Strength/Dosage Unit, Registration Number, Registration Date, and Schedule Status. Medicines were collated and named consistently where the spellings or formatting of the corresponding medicinal product varied. Only medicines appearing on the Namibian Human Medicines Register were reviewed.

### Data coding

#### Coding medicines on the NEMList for registration status

For the purpose of the study, medicines extracted from the NEMList were categorised as registered (Yes) or unregistered (No) based on whether any products with the same generic name (Active Pharmaceutical ingredient(s)/INN(s)), strength, and dosage form as appeared on the Namibian Human Medicines Register. Medicines listed on the NEMList that were not found on the Namibian Human Medicines Register were categorised as ‘unregistered’. Similarly, medicines listed on the NEMList that were found on the Namibian Human Medicines Register but differed in strength and dosage form or appeared in single or combination formulations that were different from the registered products were considered unregistered.

For example, Ascorbic Acid (Vitamin C) 100 mg tablets are listed as an essential medicine on the NEMList. However, the Namibia Human Medicines Register only has Ascorbic Acid (Vitamin C) 250 and 500 mg tablets as single Ascorbic Acid formulations, while the rest of Ascorbic Acid formulations are in combination with other medicines. As such, Ascorbic Acid 100 mg tablets were classified as ‘unregistered’ because the medicines on the Namibian Human Medicines Register did not match the Ascorbic Acid listed on the NEMList in terms of the strength of the medicine. Two independent researchers (ANA and JEKS) conducted the analysis to ensure accurate alignment between the NEMList and the Namibian Human Medicines Register in terms of the generic name of the medicines, strengths and dosage forms. Discrepancies were resolved by a third researcher (SNS).

#### Coding medicines on the Namibia Human Medicines Register for essential medicines status

The essential medicines were searched for in the Namibian Human Medicines Register to determine their registration status. A product was classified as ‘essential’ if it matched the description of the essential medicine in the NEMList in terms of the generic name of the medicine (INN(s)) as a single or fixed-dose combination, strength, and dosage form/formulation. Conversely, a registered product that did not match the medicines in the NEMList in terms of generic name, strength and dosage form/formulation was considered as ‘non-essential’.

### Data analysis

The data analysis was done as follows:

- The number and proportion of Essential Medicines in the NEMList that are registered.
- The proportion of medicines registered for each Therapeutic Class as per the NEMList.
- The number of Essential Medicines by unique INN with at least three (3) registered products. The World Health Organization recommends at least three products registered by different manufacturers per medicine to ensure a stable supply (World Health Organization, 2016). In the context of our study, a registered medicine is a medicine that has been granted marketing authorisation by the NMRC and appears on the Namibian Human Medicines Register.
- The proportion of medicines with at least three registered products for each Therapeutic Class as per the NEMList.

While there are currently no specific benchmarks for the percentage of essential medicines that should be registered at a country level, for the purpose of this study, registration proportions ≥80% were considered as ‘high registration’, registration proportions between 79% and 51% were considered as ‘Moderate’ and registration proportions below 50% were considered as ‘Low’. These interpretations were made in line with the WHO recommendations for all countries to voluntarily reach the minimum target of 80% availability of essential medicines by 2025 (Deng et al., 2023).

Additionally, a sub-group analysis was conducted for antibiotics in the NEMList. Antibiotics were sub-classified using the Access, Watch and Reserve (AWaRe) classification of antibiotics into:

- Access group antibiotics
- Watch group antibiotics
- Reserve group antibiotics

The AWaRe classification of antibiotics was developed by WHO in which antibiotics are classified into three different groups – Access, Watch and Reserve – based on their clinical importance and the risk of their use promoting resistance to emphasise the importance of their appropriate use (World Health Organization, 2023b). The Access group antibiotics have activity against a wide range of commonly encountered susceptible pathogens and a lower resistance potential (World Health Organization, 2023b). The Watch group antibiotics have higher resistance potential, are mostly critically important and/or at relatively high risk of selection of bacterial resistance, and should be prioritised as key targets of stewardship programmes and monitoring (World Health Organization, 2023). Lastly, the Reserve group antibiotics should be reserved for the treatment of confirmed or suspected infections due to multidrug-resistant organisms (World Health Organization, 2023b). The Reserve group antibiotics should be treated as ‘last resort’ options.

The data analysis for AWARE antibiotics was done as follows:

- The number and proportion of AWaRe antibiotics on the NEMList
- The number and proportion of AWaRe antibiotics on the NEMList with registered products
- The number and proportion of AWaRe antibiotics on the NEMList with at least three registered products

## Results

### Essential medicine status of products on the Namibia Human Medicines Register

Out of 5,444 registered products on the Namibia Human Medicines Register, only 23.7% (*n* = 1,288) were essential medicines (Table 1). This shows that registered products are mainly intended for the market, rather than prioritised for the public health system.

**Table 1.** Registration of essential medicines.

	*n* (%)
A) The proportion of registered medicines that are essential	
Number of registered products in the Namibia Human Medicines Register	5,444
Total number of registered products for essential medicines	1,288 (23.7%)
B) The proportion of Essential Medicines with registered products	
Overall number of medicines in the NEMList	481
Total number of registered essential medicines	386 (80.2%)
Number of essential medicines with ≥ 3 registered products	176 (45.6%)

### Registration status of essential medicines in Namibia

Out of 481 essential medicines listed on the NEMList, the majority (80.2%, *n* = 386) had at least one registered product (Table 1), suggesting generally good regulatory coverage of the NEMList.

### Essential medicines with stable supplies

Using WHO’s criterion of ≥3 products registered by different manufacturers to support supply stability, 45.6% (*n* = 176) of essential medicines met this threshold (Table 1), while 54.4% (*n* = 210) did not. This indicates that although most essential medicines are registered, their supply remains uncertain.

### Registration status of essential medicines across therapeutic classes

Table 2 summarises the registration pattern of essential medicines across Therapeutic Classes for prevalent communicable and non-communicable diseases in Namibia (see Supplemental Table S1 for all the 93 Therapeutic Classes). Just over half of the Therapeutic Classes (52.7%, *n* = 49) had at least one registered product for all their essential medicines, while 8.6% (*n* = 8) had no registered products for any of their medicines. The remaining 37.6% (*n* = 36) showed partial registration, ranging from 25.0% to 93.8%.

**Table 2.** The number and proportion of essential medicines with ≥ 3 registered products across key therapeutic classes (prevalent communicable and non-communicable diseases^a^) in Namibia.

Therapeutic class	Essential medicines per class (*n*)	Registered essential medicines per class, *n* (%)	Essential medicines with ≥3 registered products per class *n* (%)
Non-communicable diseases			
1.8 Insulins	4	4 (100%)	4 (100%)
1.9 Oral Antidiabetics	2	2 (100%)	2 (100%)
2.1 Anticoagulants	7	7 (100%)	4 (57.1%)
2.3 Anti-Anaemic Preparations	5	5 (100%)	2 (40.0%)
3.1 Cardiac Therapy	4	4 (100%)	1 (25.0%)
3.2 Cardiac Stimulants	2	2 (100%)	0 (0%)
3.3 Vasodilators Used in Cardiac Disease	3	3 (100%)	0 (0%)
3.5 Lipid Lowering Agents	3	3 (100%)	2 (66.7%)
3.6 Hypotensives	11	10 (90.9%)	7 (63.6%)
3.7 Agents Acting on Renin-Angiotensin System	2	2 (100%)	1 (50.0%)
3.8 Diuretics	6	6 (100%)	4 (66.7%)
3.9 Beta Blocking Agents	6	5 (83.3%)	4 (66.7%)
Communicable diseases			
7.6 Tuberculostatics, excluding Streptomycin	24	19 (79.2%)	11 (45.8%)
7.8 Antivirals for Systemic Use	3	2 (66.2%)	0 (0%)
7.9 Antiretrovirals	38	35 (92.1%)	21 (55.3%)
11.2 Antimalarials	4	3 (75.0%)	1 (25.0%)

^a^ World Health Organization (2023c). Country disease outlook: Namibia – August 2023. World Health Organization Regional Office for Africa. https://www.afro.who.int/sites/default/files/2023-08/Namibia.pdf (World Health Organization, 2023).

### Therapeutic classes with ≥3 registered products per essential medicine

Supply stability varies markedly across Therapeutic Classes. Only 13.9% (*n* = 13) of Therapeutic Classes had all their essential medicines supported by ≥3 registered products, while 39.8% (*n* = 37) had none (Table 2; see Supplemental Table S1 for complete list and Supplemental Table S2 for individual essential medicines). Overall, the findings highlight significant variability in both the registration and availability of essential medicines across different therapeutic categories.

### Registration of AWaRe antibiotics

Out of the 85 antibiotics on the NEMList, 64.7% (*n* = 55) fell under the WHO AWaRe classification. Among these, 47.3% (*n* = 26) were Access, 45.5% (*n* = 25) Watch and 7.3% (*n* = 4) Reserve antibiotics. Overall, 94.5% (*n* = 52/55) had registered products, including 96.2% (*n* = 25) of Access, 92.0% (*n* = 23) of Watch, and all 100% (*n* = 4) of the Reserve group antibiotics (Table 3 and Supplemental Table S3). For supply stability, 65.4% (17/26) of Access, 68.0% (17/25) of Watch and 25.0% (1/4) of the Reserve group antibiotics had ≥3 registered products.

**Table 3.** The number and proportion of AWaRe antibiotics in the NEMList with registered products.

AWaRe classification	Access antibiotics	Watch antibiotics	Reserve antibiotics
Number of antibiotics in the NEMList (*n*)	26	25	4
Total number of antibiotics with registered products *n* (%)	25 (96.2%)	23 (92.0%)	4 (100%)
Number of antibiotics with ≥ 3 registered products *n* (%)	17 (65.4%)	17 (68.0%)	1 (25.0%)UK,

## Discussion

### Essential medicines

This study explored the registration of essential medicines in Namibia as a proxy for availability of essential medicines in the country. Essential medicines accounted for only 23.7% of products on the Namibia Human Medicines Register, substantially lower than proportions reported in similar African settings, where essential medicines accounted for 29%, 37% and 42% of all registered products in Kenya, Uganda, and the United Republic of Tanzania, respectively (Green et al., 2023). This may suggest that regulatory resources in Namibia are diverted to non-essential, market-oriented products, which could potentially lead to irrational use of medicines (Green et al., 2023).

Nevertheless, 80.2% of essential medicines on the NEMList had at least one registered product, similar to findings in Kenya and Pakistan, where approximately 72% and 74% of essential medicines on the NDR were registered, respectively (Green et al., 2023; Rafi et al., 2021). However, registration varied widely across therapeutic classes. Many high-burden non-communicable disease areas such as diabetes, hypertension, high cholesterol and cardiovascular conditions showed strong coverage, with most products registered. This pattern aligns with Namibia’s growing cardiometabolic disease burden (Adekanmbi et al., 2019; Alweendo et al., 2023; Craig et al., 2018), economic burden associated with these public health issues (Bommer et al., 2018; Gnugesser et al., 2022) and the priority placed on these therapies.

Notably, registration of antiretroviral drugs (92.1%), anti-tuberculosis agents (79.2%) and antimalarial agents (75.0%) was found to be highly moderate, which could be attributed to the NMRC’s participation in the WHO collaborative procedure that enables accelerated, priority registration of prequalified finished pharmaceutical products for antiretrovirals, anti-TB and antimalarial drugs (World Health Organization, 2019), thus contributing to their wider availability in the country (World Health Organization, 2023d). The moderately high registration of these medicines in Namibia could be a significant indicator of the country’s progress and commitment towards eliminating HIV/AIDS, Tuberculosis and Malaria by 2030, as per the sustainable development goal (SDG) target 3.3 (World Health Organization, 2022).

### Supply of essential medicines

Despite the high registration of essential medicines overall, more than half of those registered (54.4%) had fewer than three products registered, indicating potential supply vulnerability. These findings are consistent with patterns in other African countries, although Namibia’s coverage is slightly better, where 38%, 45% and 36% of essential medicines had only one or two registered products in Kenya, Uganda, and the United Republic of Tanzania, respectively (Green et al., 2023).

Considerable variation existed between Therapeutic Classes and Anatomical Therapeutic Chemical (ATC) Classes, where nearly 40% of Therapeutic Classes and 60% of ATC Classes had no essential medicines supported by ≥3 products. For priority infectious disease medicines, the proportions with ≥3 registered products were low, with only 25.0% for antimalarials, 45.8% for anti-tuberculosis medicines, and 55.3% for antiretrovirals having ≥3 registered alternatives.

Additionally, while some broad classes had high registration rates, many contained multiple subclasses with limited or no supplier diversity. The most affected areas included reproductive health, oncology, dermatology, haematology, ophthalmology and ear, nose and throat (ENT), and central nervous system medicines. For example, although 77.1% of the central nervous system (CNS) medicines were registered, only 24.3% met the WHO stability threshold. Similarly, the Blood and Blood Forming Organs class had high registration (82.6%); however, only 19.6% met the stability threshold. Low-volume or specialised categories such as antipsychotics, anti-Parkinson’s agents, immunoglobulins, and many ophthalmologic preparations relied on one or two products per medicine or had none registered.

Given that WHO recommends at least three different manufacturers per medicine to ensure supply stability, these gaps could indicate potential susceptibility to shortages. A recent study by Brhlikova et al. (2025) had proposed the use of the extent to which essential medicines are registered on national drug registers to be recognised as a vital indicator of availability (Brhlikova et al, 2025), further reinforcing the importance of maintaining ≥3 registered alternatives for essential medicines to improve the availability of essential medicines. Overall, these patterns underscore the need for targeted regulatory prioritisation, particularly for low-volume but clinically essential medicines.

### Registration of AWaRe antibiotics

Of the 55 AWaRe antibiotics listed on the NEMList, three out of four had registered products. However, supply stability differed significantly across the AWaRe groups. While approximately two-thirds of Access and Watch antibiotics met the ≥3-product threshold, this was only true for one Reserve antibiotic. Given that WHO had targeted that at least 60% of total antibiotic prescribing at the country level by 2023 (World Health Organization, 2023b) and the One Health Global Leaders Group on Antimicrobial Resistance targets at least 80% of overall human antibiotic global consumption by 2030 to be Access group antibiotics (One Health Global Leaders Group on Antimicrobial Resistance, 2024); strengthening registration and supply resilience for these antibiotic groups remains critical to maintain a stable supply that can promote rational use of antibiotics.

Economically, the prioritisation of registration of Access group antibiotics could be a cost-effective strategy for the government and private sector stakeholders, given that Access group antibiotics have a lower cost compared to either Watch or Reserve group antibiotics (World Health Organization, 2023). While governments and NRAs have the option to obtain unregistered antibiotics through special import permits (e.g. Section 27 of Medicines and Related Substances Control Act 13 of 2003; Namibia Medicines Regulatory Council, 2024a; Republic of Namibia, 2003), the long-term implications for stewardship and resistance remain uncertain, which could undermine the WHO's efforts to tackle the issue of antimicrobial resistance globally. Therefore, strengthening the registration of Access antibiotics may support rational use and affordability.

### Barriers to registration of essential medicines

While our findings indicate that a high proportion of essential medicines are registered in Namibia, delays in medicine registration remain a major bottleneck in ensuring timely access to essential medicines. This is consistent with broader trends in low - and middle-income countries (LMICs), where medicine registration can take between four and seven years after dossier submission, compared to one to two years in high-income countries (Ahonkhai, 2016; Jawahar, 2018; Thomas, 2016; U.S. Food and Drug Administration (USFDA), 2019). In Namibia, persistent inefficiencies in the registration process, including prolonged evaluation timelines and significant backlogs, have impeded access to key medicines (Anisfeld et al., 2014; Mbaziira & Kagoya, 2014). Between 2011 and 2014, over 700 dossiers were awaiting evaluation, with an average of 150 new applications added annually (Anisfeld et al., 2014). Although the fast-track system had an average processing time of 53 days in 2012, the cumulative backlog continued to grow (Anisfeld et al., 2014; Mbaziira & Kagoya, 2014). These delays particularly affected the availability of newer antiretroviral (ARV) formulations, including paediatric options, thereby compromising treatment outcomes in national HIV programmes.

The challenges experienced by the NMRC in achieving its mandate are, however, not unique to Namibia. Studies into the challenges faced by other African NRAs in registering medical products have highlighted weak or absent medicines regulatory systems and capacity constraints within the region, coupled with high staff turnover, inadequate human resources, as well as incomplete or incoherent legal/regulatory frameworks (Ncube et al., 2021; Yenet et al., 2023). Collectively, these challenges underscore the need for effective strategies to strengthen the capacity of NRAs like the NMRC to register essential medicines in a timely manner, to prioritise the registration of essential medicines over non-essential medicines and to train personnel for efficient evaluation of dossiers to ensure access to and a stable supply of safe, quality and efficacious medicines.

### Strategic interventions to improve essential medicines availability

To mitigate some of the challenges faced in the timely registration of medicines, the NMRC is a member of regional medicines regulatory harmonisation bodies such as the ZAZIBONA collaborative procedure for medicines registrations, which is aimed at harmonising the medicines registration process to reduce timelines and ensure the availability of good quality medicines within the Southern African region (Tanzania Medicines and Medical Devices Authority, 2024).

Furthermore, while medicine registration is a legal prerequisite for marketing in Namibia, the country also makes use of alternative access pathways such as donations or compassionate medicine use utilising Section 27 of the Medicines and Related Substances Control Act, which allows for special authorisation of unregistered medicines. These alternative mechanisms bypass the full technical assessment required for registration, offering a temporary solution for medicine availability.

This study’s findings also highlighted the over-registration of non-essential medicines. To address this, authors of a recent study recommend routine auditing of the national drug register to assess the registration status of medicines relative to the essential medicines list (Brhlikova, Babar, & Pollock, 2025). Digitally linking the Namibian Human Medicines Register with the NEMList could enable real-time monitoring and joint decision-making between the NMRC as a regulator, the Namibia Essential Medicines List Committee and health policymakers, and could also help to identify registration gaps and inform regulatory priorities.

Local manufacturing of essential medicines also presents a viable path forward to avail essential medicines in the country and is a key priority under Namibia’s National Medicines Policy (Ministry of Health and Social Services, 2022). The WHO advocates for local manufacturing of medicines to improve supply chain resilience and equitable access (World Health Organization, 2024). To support this, Namibia could introduce targeted incentives for local and regional manufacturers to register and produce missing essential medicines (Brhlikova, Babar, & Pollock, 2025). These efforts need to be grounded in technical capacity assessments and complemented by institutional strengthening (Brhlikova, Babar, & Pollock, 2025) within the NMRC to reduce processing times and improve regulatory performance. These combined measures can enhance the NMRC’s responsiveness to allow for a stable and uninterrupted supply of essential medicines.

### Study limitations

The findings of this study should be considered in the context of its strengths and limitations. The Namibian Human Medicines Register was assessed using the data available as of February 2024 and the original NEMList seventh edition, without consideration of any requests for changes (deletions or additions) to the NEMList that may have taken place after the official launch of the physical document in 2021. Updates to the Namibian Human Medicines Register after February 2024 could not be considered. The differences in methodologies adopted in our study, such as only considering full strength per registered product and not half-strength products compared to similar studies, may impair the comparability of our findings with the results of similar studies. Moreover, the study did not examine the actual availability of essential medicines at the Central Medical Stores or public health facilities; we used regulatory registration as a proxy for medicine availability. However, registration alone does not ensure procurement, supply, or presence at points of care. As such, there may be potential mismatches between the registration of essential medicines and the access to these medicines in the country. Therefore, registration may not reflect overall access, which depends on multiple determinants beyond the scope of this study . Furthermore, the current study did not assess the affordability of essential medicines in Namibia, a key consideration in assessing the access to medicines in the country. Nevertheless, this study paints a better picture of the registration of essential products in Namibia compared to other countries, despite the challenges faced by the NMRC and the application of the stringent registration processes.

## Conclusion

To the best of our knowledge, this is the first study assessing the registration status of essential medicines in Namibia. This study found that the majority of medicines on the Namibian Human Medicines Register are non-essential medicines. Achieving the WHO’s recommendation of at least three alternative products per essential medicine remains difficult under the current regulatory conditions. Further research is necessary to identify the bottlenecks encountered by the NMRC in the registration of medicines, especially essential medicines. Moreover, it is pertinent to understand factors that are contributing to the exploration of other avenues to avail medicines in the country such as the use of section 27 of the MRCSA, donations and pooled procurement by the government, their limitations, and benefits. Collectively, the findings of our study highlight the need for concerted efforts between the NMRC and other stakeholders to strengthen regulatory capacity for registering essential medicines and to reduce the variability in the registration of medicines across therapeutic classes. Furthermore, the study provides an opportunity for the NMRC to re-assess its registration priorities, in the context of registration of essential medicines and resource utilisation, repurposing and/or prioritisation to ensure a stable and sufficient availability of essential medicines in Namibia.

## Supplementary Material

Supplemental Material
