# Supplementary material for: An analysis of the registration of essential medicines in Namibia: a proxy measure of the availability of essential medicines
Source: J Pharm Policy Pract. 2026 Mar 23;19(1):2643322. doi: 10.1080/20523211.2026.2643322 (PMC13011098; doi:10.1080/20523211.2026.2643322)
Supplement: Supplemental Material [file JPPP_A_2643322_SM3606.docx]

An analysis of the registration of essential medicines in Namibia: A proxy measure of the availability of essential medicines

**Supplementary Online Resources [Additional Files]**

**Supplementary Table S1. Proportion of registered Essential Medicines in the NEMList with ≥ 3 Products per International Anatomical Therapeutic Chemical (ATC) Class**

| **Anatomical Therapeutic Chemical (ATC) classification system** | **Total Products (N)** | **Registered Products (n)** | **% Registered Products (%n)** |
| --- | --- | --- | --- |
| Musculoskeletal System | 9 | 9 | 100 |
| Respiratory System | 12 | 12 | 100 |
| Cardiovascular System | 39 | 37 | 94.9 |
| General Anti - Infectives. Systemic | 149 | 127 | 85.2 |
| Systemic Hormones, excl. Sex Hormones | 12 | 10 | 83.3 |
| Blood & Blood Forming Organs | 46 | 38 | 82.6 |
| Alimentary Tract & Metabolism | 49 | 39 | 79.6 |
| Central Nervous System | 70 | 54 | 77.1 |
| Antiparasitic Products | 12 | 9 | 75 |
| Dermatologicals | 28 | 18 | 64.3 |
| Genito-Urinary System & Sex Hormones | 22 | 14 | 63.6 |
| Sensory Organs (Ophthalmologicals & Otologicals) | 21 | 13 | 61.9 |
| Various | 11 | 6 | 54.5 |
| Antineoplastic & Immunosuppressives | 1 | 0 | 0 |
| **TOTAL** | **481** | **386** | **80.2** |

**Supplementary Table S2.** **Proportion of Essential Medicines in the NEMList by Unique INN with ≥ 3 registered products per International Anatomical Therapeutic Chemical (ATC) Class**

| **Anatomical Therapeutic Chemical (ATC) classification system** | **Total Medicines (N)** | **Medicines with ≥3 registered Products (n)** | **% Medicines with ≥3 registered Products (%n)** |
| --- | --- | --- | --- |
| Musculoskeletal System | 9 | 6 | 66.7 |
| Respiratory System | 12 | 6 | 50 |
| Cardiovascular System | 39 | 19 | 48.7 |
| General Anti - Infectives. Systemic | 149 | 70 | 47 |
| Alimentary Tract & Metabolism | 49 | 23 | 46.9 |
| Dermatologicals | 28 | 11 | 39.3 |
| Antiparasitic Products | 12 | 4 | 33.3 |
| Genito-Urinary System & Sex Hormones | 22 | 6 | 27.3 |
| Systemic Hormones, excl. Sex Hormones | 12 | 3 | 25 |
| Central Nervous System | 70 | 17 | 24.3 |
| Blood & Blood Forming Organs | 46 | 9 | 19.6 |
| Sensory Organs (Ophthalmologicals & Otologicals) | 21 | 2 | 9.5 |
| Antineoplastic & Immunosuppressives | 1 | 0 | 0 |
| Various | 11 | 0 | 0 |
| **TOTAL** | **481** | **176** | **36.6** |

**Supplementary Table S3. Registration of AWaRe Antibiotics in the NEMList and AWaRe Antibiotics with ≥ 3 Registered Products**

| **Antibiotics in the NEMList** | **AWaRe Class** | **Registered (Yes/No)** | **≥ 3 registered products** |
| --- | --- | --- | --- |
| Cefazolin 1 gram Powder for Injection | Access | Yes | Yes |
| Amoxycillin 250mg/5ml Syrup |  | Yes | Yes |
| Amoxycillin 500mg Capsules |  | Yes | Yes |
| Amoxycillin + Clavulanic Acid 125mg + 31mg Suspension |  | Yes | Yes |
| Amoxycillin + Clavulanic Acid 1000 + 200 mg Injection |  | Yes | Yes |
| Amoxycillin + Clavulanic Acid 875 + 125mg Tablets |  | Yes | Yes |
| Ampicillin 250mg Powder for Injection |  | Yes | Yes |
| Ampicillin 500mg Powder for Injection |  | Yes | Yes |
| Benzathine Benzylpenicillin 2.4 million IU Powder for Injection |  | Yes | No |
| Benzylpenicillin 1 million IU Powder for Injection |  | Yes | No |
| Cloxacillin 500mg Capsules |  | Yes | No |
| Cloxacillin 250mg/5ml Syrup |  | No | No |
| Cloxacillin 250mg Powder for Injection |  | Yes | Yes |
| Cloxacillin 500mg Powder for Injection |  | Yes | Yes |
| Phenoxymethylpenicillin 250mg Tablets |  | Yes | No |
| Phenoxymethylpenicillin 250mg/5ml Syrup |  | Yes | Yes |
| Chloramphenicol 1g Powder for Injection |  | Yes | No |
| Clindamycin 150mg Capsules |  | Yes | Yes |
| Clindamycin 150mg/ml Injection (4ml) |  | Yes | No |
| Doxycycline 100mg Capsules |  | Yes | Yes |
| Gentamicin 10mg/ml Injection (2ml) |  | Yes | Yes |
| Gentamicin 40mg/ml Injection (2ml) |  | Yes | Yes |
| Nitrofurantoin 100mg Capsules |  | Yes | No |
| Sulfamethoxazole + trimethoprim (Co-trimoxazole) 80mg + 400mg Tablets |  | Yes | Yes |
| Sulfamethoxazole + trimethoprim (Co-trimoxazole) (40mg + 200mg) /5ml Suspension |  | Yes | Yes |
| Sulfamethoxazole + trimethoprim (Co-trimoxazole) (80mg + 400mg) /5ml Injection |  | Yes | No |
| Cefixime 200mg Tablets | Watch | Yes | No |
| Ceftriaxone 250mg Powder for Injection |  | Yes | Yes |
| Ceftriaxone 1g Powder for Injection |  | Yes | Yes |
| Cefuroxime 750mg Powder for Injection |  | Yes | Yes |
| Cefuroxime 500mg Tablets |  | Yes | Yes |
| Cefuroxime 125mg/5ml Suspension |  | Yes | No |
| Piperacillin + Tazobactam 4g + 500mg Powder for Injection |  | Yes | Yes |
| Azithromycin 500mg Tablets |  | Yes | Yes |
| Azithromycin 200mg/5ml Suspension |  | Yes | Yes |
| Ciprofloxacin 500mg Tablets |  | Yes | Yes |
| Ciprofloxacin 2mg/ml Infusion (200ml) |  | Yes | Yes |
| Erythromycin 125mg/5ml Suspension |  | Yes | Yes |
| Erythromycin 250mg Tablets |  | Yes | No |
| Meropenem 500mg Injection |  | Yes | Yes |
| Meropenem 1g Injection |  | Yes | Yes |
| Streptomycin 1g Powder for Injection |  | Yes | No |
| Vancomycin 500mg Injection |  | Yes | Yes |
| Vancomycin 1g Injection |  | Yes | Yes |
| Clarithromycin 500mg Tablets |  | Yes | Yes |
| Levofloxacin 250mg Tablets |  | Yes | Yes |
| Moxifloxacin 400mg Tablets |  | Yes | Yes |
| Moxifloxacin 400mg/250ml Infusion |  | Yes | No |
| Rifampicin 150mg Capsules |  | Yes | No |
| Rifampicin 450mg Tablets |  | No | No |
| Rifampicin 100mg/5ml Syrup |  | No | No |
| Fosfomycin 3g Powder for Injection | Reserve | Yes | No |
| Linezolid 2mg/ml Infusion (300ml) |  | Yes | No |
| Linezolid 400mg Tablets |  | Yes | No |
| Linezolid 600mg Tablets |  | Yes | Yes |
